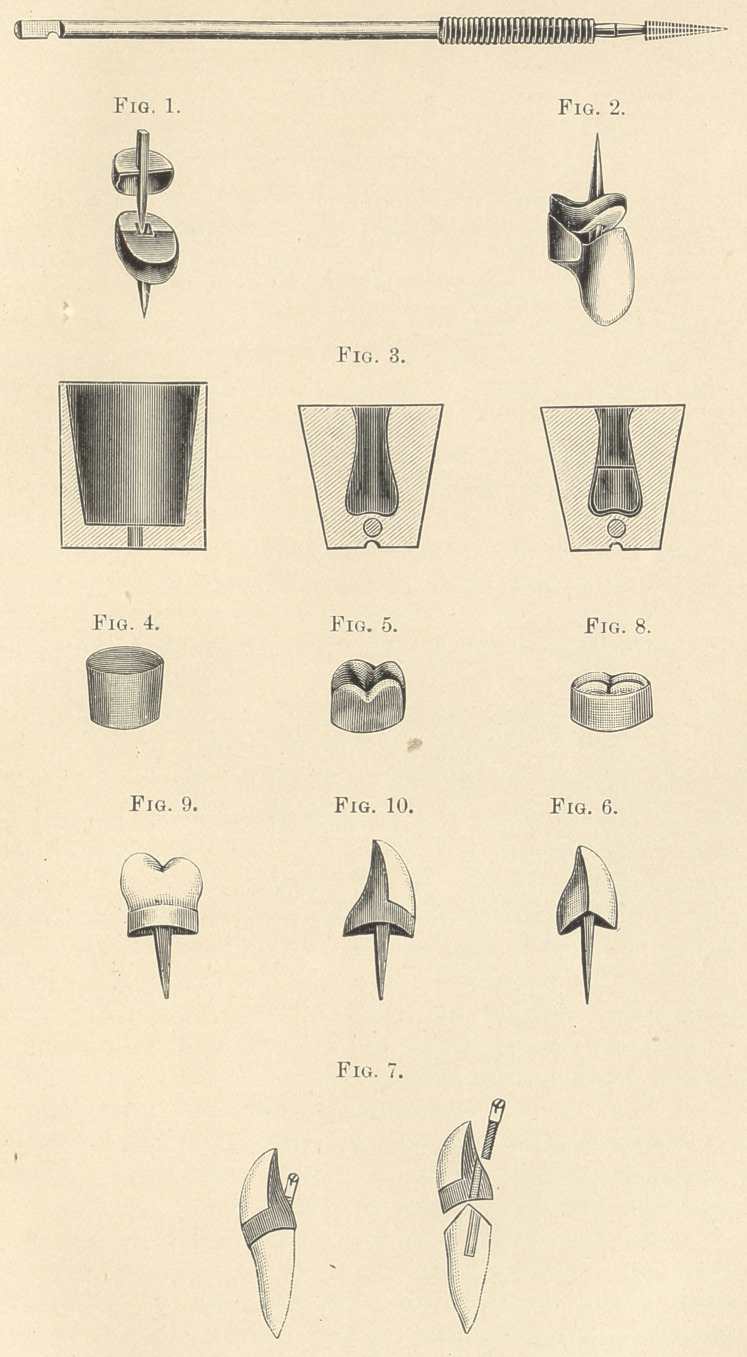# Crown- and Bridge-Work

**Published:** 1893-02

**Authors:** C. M. Richmond

**Affiliations:** New York


### International Dental Journal.

Vol. XIV. February, 1893. No. 2.

### Original Communications.[1]

### CROWN- AND BRIDGE-WORK.[2]

BY DR. C. M. RICHMOND, NEW YORK.

(Continued from Vol. XIII., page 860.)

In this article is given the first of a series on crowns. I have illustrated an instrument which I have devised for the purpose of cutting or enlarging the cavity in roots of teeth to receive a post or tube as may be desired. All pins and all tubes are made square and tapering, and with this instrument the canal can be cut to the size, so the pin or tube will fit into it and the corners will touch the tooth the entire depth of the canal. It will be seen that a pin or tube could be made to hold a crown without the aid of a band or cement, so perfect is it possible to fit by this method. I had this instrument mounted on a short cable, as it at once adjusts itself to the direction of the canal, and the disagreeable sensation produced by a rigid instrument is avoided. In making the square tapering pin, a piece of round wire of irido-platina is used. The end is first hammered nearly to a taper form, and by this method the wire becomes harder and stiffer than in any other way. It is now filed to a taper as nearly perfect as possible. I have not found square wire as hard and rigid

[1] The editor and publishers are not responsible for the views of authors of papers published in this department, nor for any claim to novelty, or otherwise, that may be made by them. No papers will be received for this department that have appeared in any other journal published in the country.

[2] Copyrighted, 1892, by Dr. C. M. Richmond.

as wire made this way. Tubes are made for the pin by cutting a piece of twenty-two-carat gold the proper taper, so that when it is rolled around the pin it will come together and lap slightly, and in rolling or bending the first angle is made with a pair of flat-nose parallel pliers, using a hammer to get the first bend at a perfect right angle the whole length of the gold strip. The tapering pin is now placed into the gold, and, holding it in position at the first angle with pliers, I bend the second angle, and so on until a square tapering tube is prepared, and one that nearly fits the pin it is made over. I wish to solder this tube where it laps, and to do this without the least solder flowing inside and so ruining it, I file the lapping surfaces down to a straight edge and also quite thin, as I cover this surface with another piece of gold, and so thicken and strengthen it in turn. A thin, flat piece of plate is taken as long and a trifle wider than the tube, and on it is placed a small piece of solder, and with the blow-pipe I blow this the entire length. The tube is placed with the side which is lapped and filed down on the surface of the plate where we have our solder. Gradually heat to the soldering-point, and have so much surface to solder on the filed surface of the tube that when this is done there is no solder to run into the tube, it being all used in soldering the tube and plate together. Half of the tubes I tried to make were ruined until I hit upon this method, and I have not made an imperfect one since this plan has been used, and have frequently had students who would make them and get them right the first time. I now place the pin in the tube, and try it until I find which way it fits best, as no two sides of a tapering pin made with a file are alike, and the pin must be put into the tube the way it was made to go, and the pin and tube must be marked with a file so as to be sure not to get it together wrong, as a pin made to go one way and then turned half around would not go into place when the crown is finished. After the tube is soldered, the pin is driven into it, and with the hammer is filled, driving it until it is perfect.

In Fig. 1 will be seen the pin-and-tube combination. I make and fit a gold-cap as in my band-crown, cutting a square hole in the centre or opposite the canal in the root, the cap is put on the root, and the tube is put through the hole previously cut, and is carried down to its place in the root. The two are now waxed together with hard wax and again tried on. In placing the tube in its proper position, I place the side previously marked towards the front or facial surface, as the mark will disappear in soldering the tube into the cap. After the tube and cap are as we wish to solder them, the cap is in-

Fig. 1.

Fig. 2.

Fig. 3.

Fig. 4.

Fig. 5.

Fig. 8.

Fig. 9.

Fig. 10.

Fig. 6.

Fig. 7.

vested, and a small amount of investment is also placed into the mouth of the tube to prevent any solder creeping into it while soldering. After the tube and cap are soldered together, they are placed in acid until the investment is entirely cleaned out of the tube, which takes some time. The top of the tube is now filed down to the cap, as shown in the lower half of Fig. 1. The pin is now placed into the tube, and the marked side is placed towards the facial surface, as it was made to go.

A piece of gold is cut to just fit the half-cap, and a piece of gold bent (which is crescent-shaped) to go on to the palatal surface of the half-cap, as shown in Fig. 1. This is waxed together,—pin, half cap, and crescent band of gold,—and invested and soldered. We now have finished the crown or gold parts as shown in Fig. 1. I place this on the root, grind the tooth into its proper position, wax it on to the pin, and invest and solder as for any crown; and it is shown finished in Fig. 2. This is the crown I use in movable bridges, and, if it is properly made, it holds a bridge as firmly as a fixed piece. Care in all of the detail is required, as absolute perfection must be obtained or a failure is sure to result.

I show a system of constructing all gold crowns of one piece of metal, and not seamless.

Fig. 3 is a metal matrix and shell for holding the same. I use a steel plunger of the size of the entrance into the matrix. A piece of gold is cut large enough to go around the plunger, and is bent and soldered; I leave sufficient length of material to slit the top, and bend the pieces of gold over the end of the plunger, which makes a shapeless crown, like Fig. 4. This is now filled with dry marble-dust, placed in my matrix, put into the holding-shell, and with one blow from a hammer a crown is produced, which is a perfect *fac-simile* of any matrix which I use. The seams are now soldered, which stiffens the top and makes it very durable. After polishing, the seams are not perceptible, and the crown can be fitted to any case applicable. These dies will soon be in the market, and will fill a place in the laboratory as a time-saver.

Fig. 4 exhibits the shapeless crown, and Fig. 5 shows it after swaging with the marble-dust. These dies can be sold at fifty cents each; and all sizes and shapes of gold crowns can be produced in the laboratory for less than one dollar each, average cost.

Fig. 6 shows a crown, which I term V-shape. The roots are bevelled at the gum line, both at the facial and palatal edge; the canal is cut and enlarged as for any crown. A square tapering pin is fitted in the way described; a piece of pure gold thirty thick is cut the size and shape of the end of the root; a hole is cut in the centre smaller than the pin, which is pushed with a pair of flat-nose pliers through the gold and into the root, the pin cutting its way through the gold as it is carried to its place in the canal. The pin now holds the gold in place, and with a burnisher of the right shape the gold is burnished on to the two bevelled surfaces of the root. The pin is now withdrawn, the gold being removed with it, and it is then invested and a small piece of solder placed on it, and the gold and pin soldered together. It is then cleaned with acid and placed again on the root, and the gold is again burnished perfectly to the root, and the edges, if they overlap, are carefully trimmed off. We now have a cap of gold perfectly fitting the end of the root, and a square pin fitting the root, as described. I place this in position on the root, and grind a plain plate tooth to fit the bevel while in the mouth. After doing this, the pin and cap are removed, and the tooth waxed on with hard wax. It is now tried on the root again and adjusted, then invested and the tooth soldered to the gold cap and pin. A small pellet of gutta-percha is warmed and flattened out very thin. A piece is cut the size of the end of the root, a hole made in the centre, and the piece slipped on to the pin up to the crown. It is then warmed in a spirit flame until sufficiently soft to adjust itself perfectly to the root. It is pressed home on the root while warm, and allowed to cool. It is then removed and the surplus trimmed off with a sharp knife after slightly heating the blade. The root is dried, and with a thin spatula some cement is carried to the end of the canal in the root, and a small amount of cement is placed on the pin and gradually carried to its place on the root. I consider this crown superior to all known methods of adjusting single crowns, and give it preference to the band-crown, as it is universal in its application, and will be a success where a band would not be tolerated. All temperaments will not act kindly to a band under the gum-tissues, while all can wear this style without trouble if they can endure anything on a root. The square and perfectly-fitting pin and the V-shape combination make it impossible to split the root or to turn on the root, while the joint is made impervious to moisture and is also absolutely cleanly.

In Fig. 7 I have illustrated the first of band-crowns demonstrated by myself in New York fifteen years ago, and called the screw crown. The crown is made like all band-crowns, with the exception of the post or pin. After the crown is ready to cement, a hole is cut through the palatal surface, and the bevelled screw-head is fitted into it, with a small long burr made the same size and taper as the screw-head. After this is done, the nut or inside screw is adjusted by screwing the outer screw into it, the canal being previously prepared, the crown is tried on to the root, and if it lets the inside screw deep enough and the crown is in its proper position on the root, it is removed, and enough cement to fill the canal is mixed, and it is carried into place, and the crown again placed in position and the screw pressed down into the cement, which is allowed to thoroughly crystallize. The outer screw is unscrewed with a screw-driver, and the nut or minor screw is left embedded in the cement in the root. The root is dried again, and a small amount of cement is placed in the band of the crown, the screw started into the one in the root, the crown carried to its place, and the screw set up where it belongs with a screw-driver. The part of the screw that projects is polished off with a smoothing-stone and engine, and is finished. Should this crown need repairing at any time, the screw-head can be cut out and the crown removed easily, and by unscrewing the first screw a new one is used for resetting.

Fifteen years ago I set a crown like this at a clinic at S. S. White's Dental Depot. It is still perfect, showing the durability of this style of crowns.

Fig. 8 shows a band molar with a porcelain top. This band is made of gold- and platina-plate combined,—gold on one side and platina on the other,—for, as I wished to fuse porcelain into it, platina is required, as the heat sufficient to melt the tooth-substance would also melt the gold. After the band was fitted to the root, a piece of wax was placed inside the band crowded down to the root, to determine where to place a piece of platina to separate the root from the porcelain, and also to make the band strong by means of this centre piece. A model of investment was run into the band and into the wax which was placed there while the band was on the root. After removing the wax, I fitted a piece of the same metal, that the band was made of, inside of the band and down to the place the root came, indicated by the investment. This was then soldered, put into acid, and cleansed, and we had a band with a centre-piece soldered about midway of the same. I mixed some of the porcelain which is used in glass fillings, and filled up the crown part and carved into shape of a molar. This was placed on the fire and dried, and brought to the proper heat for the blow-pipe, which was now used, and the crown fused into the top of the band. As the first shrinks some, a second building is done, and after heating over a slow fire, the blow pipe is used to finish, and a crown is produced which is preferable to all gold if it is to be placed in the mouth of one whose teeth are prominent while speaking or laughing. I make this crown and place it on the root at one sitting, or as quickly as an all-gold crown can be made and adjusted. It was first suggested by Dr. J. Bond Littig, of New York.

Fig. 9 shows a bicuspid crown, with band and pin combination. The tooth used in this operation is the countersunk style, with a pin baked in the space at the neck. In constructing this style of crown I first fit the band to the root, and, after selecting the tooth required, it is ground to fit inside of the projecting end of the gold band. This is done out of the mouth. After the band and tooth have been ground together, it is tried on the root and adjusted. A piece of pure gold is now cut to fit inside of the band, which goes over the root, making a hole for the pin. The piece of gold is worked down in contact with the tooth-substance, and it also fits the band and pin. The tooth is now invested tooth down, and the pin and band are exposed. I heat up to the proper point before using the blow-pipe, which is used in forcing enough solder into the band to attach pin, band, and all together. This makes a very artistic piece of work and strong as well.

Fig. 10 shows the oft-illustrated band-crown, and is constructed as before described.

(To be continued.)

## Figures and Tables

**Fig. 1. Fig. 2. Fig. 3. Fig. 4. Fig. 5. Fig. 6. Fig. 7. Fig. 8. Fig. 9. Fig. 10. f1:**